# Progress towards reduced-crude protein diets for broiler chickens and sustainable chicken-meat production

**DOI:** 10.1186/s40104-021-00550-w

**Published:** 2021-03-08

**Authors:** Sonia Yun Liu, Shemil P. Macelline, Peter V. Chrystal, Peter H. Selle

**Affiliations:** 1 Poultry Research Foundation within The University of Sydney, Camden Campus, 425 Werombi Road, Camden, NSW 2570 Australia; 2 School of Life and Environmental Sciences, The University of Sydney, Camperdown, NSW 2006 Australia; 3 Baiada Poultry Pty Limited, Pendle Hill, NSW 2145 Australia; 4 Sydney School of Veterinary Science, The University of Sydney, Sydney, NSW 2006 Australia

**Keywords:** Amino acids, Broiler chickens, Glucose, Insulin, Protein, Starch, Threonine

## Abstract

The prime purpose of this review is to explore the pathways whereby progress towards reduced-crude protein (CP) diets and sustainable chicken-meat production may be best achieved. Reduced-CP broiler diets have the potential to attenuate environmental pollution from nitrogen and ammonia emissions; moreover, they have the capacity to diminish the global chicken-meat industry’s dependence on soybean meal to tangible extents. The variable impacts of reduced-CP broiler diets on apparent amino acid digestibility coefficients are addressed. The more accurate identification of amino acid requirements for broiler chickens offered reduced-CP diets is essential as this would diminish amino acid imbalances and the deamination of surplus amino acids. Deamination of amino acids increases the synthesis and excretion of uric acid for which there is a requirement for glycine, this emphasises the value of so-called “non-essential” amino acids. Starch digestive dynamics and their possible impact of glucose on pancreatic secretions of insulin are discussed, although the functions of insulin in avian species require clarification. Maize is probably a superior feed grain to wheat as the basis of reduced-CP diets; if so, the identification of the underlying reasons for this difference should be instructive. Moderating increases in starch concentrations and condensing dietary starch:protein ratios in reduced-CP diets may prove to be advantageous as expanding ratios appear to be aligned to inferior broiler performance. Threonine is specifically examined because elevated free threonine plasma concentrations in birds offered reduced-CP diets may be indicative of compromised performance. If progress in these directions can be realised, then the prospects of reduced-CP diets contributing to sustainable chicken-meat production are promising.

## Introduction

There is a genuine quest to develop effective, reduced crude protein (CP)-diets for broiler chickens because their acceptance would generate several material advantages. Certainly, some progress has been realised; nevertheless, several intriguing open questions remain and if they were to be answered the acceptance of reduced-CP diets would be accelerated to the advantage of sustainable chicken-meat production.

Consideration to the compelling justification for this objective has been provided by Greenhalgh et al. [1]. Reduced-CP broiler diets are environmentally advantageous in that they mitigate nitrogen (N) and ammonia (NH_3_) emissions and N pollution is of increasing importance, particularly in Europe. Reduced-CP broiler diets improve litter quality and reduce the incidence of foot-pad lesions and related conditions, which is beneficial for bird welfare. For example, van Harn et al. [2] reduced dietary CP contents of diets based on maize, wheat and soybean meal by up to 30 g/kg. This strategy significantly increased litter dry matter but reduced total nitrogen concentrations in litter and enhanced food pad scores from 143 to 39 units. Reduced-CP broiler diets attenuate flows of undigested protein into the hind-gut to fuel the proliferation of potential pathogens to the advantage of flock health [3]. Importantly, in addition, reduced-CP broiler diets have the potential to diminish the chicken-meat industry’s soybean meal requirements substantially, because non-bound amino acids may be construed as an alternative ‘protein’ source to soybean meal [4]. Moreover, a reduction in the industry’s dependence on soybean meal should result in less neo-tropical deforestation in South America in order to harvest soybeans [5], which is a further ecological advantage. The likelihood is that non-bound amino acid inclusion costs will become increasingly viable from economies of scale in their production which, coupled with increasing soybean meal prices, will encourage the adoption of reduced-CP diets into the future.

Background to the development of reduced-CP diets has been documented by Chrystal et al. [6]. Essentially, a reduced-CP diet is one with increased feed grain inclusions but decreased inclusions of protein-rich feedstuffs including soybean meal; however, selected amino acid targets are met by escalated inclusions of non-bound (synthetic, crystalline, feed-grade) amino acids. One consequence of these formulation modifications is that dietary starch concentrations increase and dietary starch:protein ratios expand, which may have negative consequences. Nevertheless, moderate reductions of up to 30 g/kg CP are quite feasible but more tangible reductions usually compromise feed conversion ratios (FCR) with associated increases in fat deposition which may be monitored by relative fat-pad weights.

The impacts of reducing dietary CP contents on apparent digestibility coefficients of amino acids are discussed in this review as are amino acid imbalances, deamination of surplus amino acids and the possibility that this may result in excessive levels of ammonia. The relative merits of maize and wheat as the basis of reduced-CP diets are compared against the background of starch-protein digestive dynamics. Finally, consideration is given to glycine and serine, or glycine equivalents, and threonine is specifically examined, especially in relation to free threonine plasma concentrations. The primary objective of this critical review is to consider the pathways by which progress towards reduced-CP diets and sustainable chicken-meat production may be best realised.

## Amino acid digestibilities

It is our contention that the impacts of dietary CP reductions on the apparent digestibility coefficients of amino acids have not received adequate attention. The paucity of amino acid digestibility assays in reported reduced-CP diet feeding studies may reflect an unstated assumption that dietary CP reductions have little or no impact on amino acid digestibilities; if so, the assumption is not valid. An overall average increase of 6.05% (0.877 versus 0.827) in the ileal digestibility of 17 amino acids at 21 and 42 days post-hatch following CP reductions of 27 g/kg in grower and finisher maize-based diets has been reported [7]. Hilliar et al. [8] reported an average decrease of 3.63% (0.823 versus 0.854) in digestibility coefficients of 16 amino acids in birds offered wheat-based diets at 21 days post-hatch following a CP reduction from 227 to 191 g/kg. In a second experiment, Hilliar et al. [9] found an average increase of 9.10% (0.851 versus 0.780) in 16 amino acids in birds offered 200 and 170 g/kg CP, wheat-based diets at 35 days post-hatch where the increases were statistically significant for 12 amino acids. However, the outcomes at 21 days post-hatch in the same study were quite different; amino acid digestibility coefficients of eight amino acids were increased but decreased across the eight remaining amino acids assessed, although most of these differences were numerical. Thus, it is quite evident from the three studies mentioned that reducing dietary CP levels does impact on amino acid digestibilities.

The Poultry Research Foundation has now completed eight amino acid digestibility assays in maize- and wheat-based diets as summarised in Table 1. The outcomes following dietary CP reductions are variable and the relevant papers [10–15] (Chrystal PV, Greenhalgh S, McInerney BV, McQuade LR, Selle PH, Liu SY: Maize-based diets are more conducive to crude protein reductions than wheat-based diets for broiler chickens, submitted) may be examined for specific details. Mean percentage responses ranged from a decrease of 8.21% to an increase of 29.4% in distal jejunal digestibility coefficients. In the distal ileum, mean percentage responses ranged from a decrease of 8.36% to an increase of 7.43%. The tabulated outcomes emphasise the core finding that dietary CP reductions generate fluctuations in amino acid digestibility coefficients. Increases in amino acid digestibility coefficients pursuant to reductions in dietary CP can be explained in part by the notional 100% digestibility of non-bound amino acids [16]. It is also probable that reductions in dietary CP attenuate endogenous amino acid flows [17], which would increase their apparent digestibility coefficients. Alternatively, pronounced decreases in digestibility coefficients were observed in maize-based diets [10] where overall amino acid digestibility coefficients were reduced by 8.36% in the distal ileum. This study was unusual in that the reduced-CP diets contained high quantities of maize starch; moreover, negative linear relationships were detected between ileal starch digestibility (which increased) and the digestibilities of 11 from a total of 16 amino acids. This outcome may suggest that glucose and amino acids were competing for intestinal uptakes through co-absorption with sodium (Na) via through their respective Na^+^-dependent transport systems. In addition, amino acids may compete amongst themselves for intestinal uptakes [18, 19] via numerous Na^+^-dependent and Na^+^-independent transporters with overlapping specificities [20].

**Table 1:** Impacts of dietary CP reductions on average (*n* = number of amino acids) apparent jejunal and ileal amino acid digestibility coefficients in broiler chickens offered maize- and wheat-based diets

Feed grain, *n*	CP reduction, g/kg	Distal jejunum			Distal ileum			Reference
		Coefficients	Response, %	Range, %	Coefficients	Response, %	Range, %	
Maize, 16	213 to 172	0.658 to 0.604	−8.21	−13.2 to −1.96	0.801 to 0.734	−8.36	−15.1 to − 2.67	[8]
Wheat, 16	215 to 165	0.594 to 0.600	+ 1.01	− 16.0 to + 12.2	0.748 to 0.721	− 3.61	− 14.8 to + 2.13	[9]
Wheat, 16	213 to 197.5	-	-	-	0.740 to 0.795	+ 7.43	+ 3.19 to + 14.2	[10]
Maize, 17	210 to 165	0.459 to 0.594	+ 29.4	+ 16.8 to + 51.8	0.744 to 0.790	+ 6.18	+ 2.73 to + 10.8	[11]
Maize, 16	210 to 165	0.693 to 0.758	+ 9.38	+ 1.93 to + 34.5	0.792 to 0.797	+ 5.98	− 0.43 to + 21.2	[12]
Maize, 16	208 to 165	0.617 to 0.665	+ 7.78	−0.29 to + 19.6	0.789 to 0.776	− 1.65	− 7.41 to + 4.73	[13]
Maize, 15	222 to 165	-	-	-	0.797 to 0.771	− 3.87	− 32.2 to + 15.4	[14]
Wheat, 15	222 to 165	-	-	-	0.757 to 0.736	− 2.77	− 62.7 to + 14.5	[14]

**Fig. 1:**
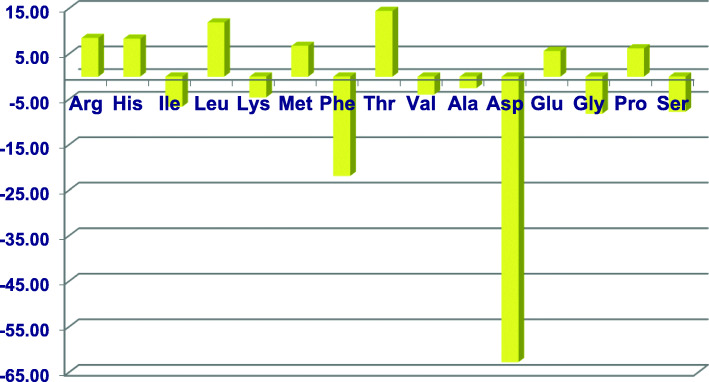
Percentage increases and decreases in apparent ileal amino acid digestibility coefficients following CP reductions from 222 to 165 g/kg in maize-and wheat-based diets. Adapted from Chrystal et al. [14]

Thus, apparent amino acid digestibility coefficients may be either amplified or compromised by reductions in dietary CP. This divergence was unequivocally demonstrated by Chrystal et al. (Chrystal PV, Greenhalgh S, McInerney BV, McQuade LR, Selle PH, Liu SY: Maize-based diets are more conducive to crude protein reductions than wheat-based diets for broiler chickens, submitted) in both maize and wheat-based diets with CP contents of 222 and 165 g/kg. Ileal amino acid digestibility coefficients of seven amino acids were increased to a significant extent but seven amino acids were significantly decreased. Alanine was the only amino acid that was not impacted by the reduction in dietary CP as illustrated in Fig. 1.

The situation is complex. Non-bound amino acids are absorbed more rapidly than their protein-bound counterparts [21] but the balance of non-bound to protein-bound amino acids increases as dietary CP levels are reduced. Moreover, the digestion of ‘intact’ proteins, including soy protein, yields di- and tri-peptides or oligopeptides, which are principally absorbed via the oligopeptide transporter, PepT-1 [22]. The salient point is that substantially more amino acids are absorbed as oligopeptides than as single amino acids [23] and di- and tri-peptides are absorbed more rapidly and efficiently than single or non-bound amino acids [24, 25]. Nonetheless, as dietary CP levels are reduced they contain less intact proteins with the capacity to yield oligopeptides. This may be an obstacle to the development of reduced-CP diets as the extent to which non-bound amino acids can replace intact proteins in broiler diets may be limited by more efficient intestinal uptakes of oligopeptides.

## Amino acid imbalances

Dietary CP reductions generate tangible fluctuations in digestibilities and intestinal uptakes of amino acids which have the potential to prompt amino acid imbalances. Interestingly, it has been suggested that amino acid imbalances are more likely to occur in low protein diets [26]. However, these fluctuations are almost certainly compounded by further variations in the post-enteral availability of amino acids. Intestinal uptakes of amino acids do not dictate their post-enteral availability because, rather than directly entering the portal circulation, substantial quantities of amino acids may enter either catabolic or anabolic pathways within enterocytes to generate energy to drive digestive processes or to synthetise proteins, including mucin, and other metabolites [27, 28].

It has been suggested that depressed feed intakes in rats may be triggered by appetite-regulating regions in the brain detecting post-enteral amino acid imbalances [29]. However, it is possibly more likely that excessive plasma ammonia levels impact on the prepyriform cortex in rats to depress feed intakes [30, 31]. Thus, it is possible that excessive plasma ammonia levels, arising from imbalances and deamination of surplus amino acids, can impede growth performance. It is then relevant that increasing systemic plasma ammonia concentrations in broiler chickens have been associated with compromised growth performance in some studies [32–34]. Plasma ammonia concentrations may be volatile over the time it takes to collect blood samples from poultry [35] and this may have a confounding effect. Alternatively, uric acid concentrations in systemic plasma were determined in broiler chickens offered diets with two CP levels and four threonine concentrations from 22 to 42 days post-hatch [36]. There is a strong indication in this study that elevated uric acid plasma concentrations were associated with depressed feed intakes.

## Costs of deamination

Amino acid imbalances give rise to a surplus of amino acids which are catabolised [37], and this invites a consideration of the costs of deamination and the possibility of excessive levels of ammonia being generated [38]. The likely genesis of amino acid imbalances in birds offered reduced-CP diets includes disparities in digestibility coefficients, different digestive kinetics of non-bound versus protein-bound amino acids and the possibility that non-bound amino acids are more likely to be spared in their transition across the gut mucosa. In the main, surplus amino acids undergo oxidative deamination in the liver which generates ammonia that requires detoxification [39]. Ammonia is detoxified by an energy-consuming, condensation reaction catalysed by glutamine synthetase in which ammonia and glutamic acid are converted into glutamine as shown in the following equation [40]:

$$ {{\mathrm{NH}}_4}^{+}+\mathrm{Glu}+\mathrm{ATP}+{\mathrm{Mg}}^{2+}\Rightarrow \mathrm{Gln}+\mathrm{ADP}+\mathrm{Pi}. $$

The entry of glutamine into the Krebs uric acid cycle allows the nitrogen (N) component of ammonia arising from deamination to be ultimately excreted as uric acid-N. There is an obligatory glycine input into the Krebs uric acid cycle where one mole of glycine is required for every mole of uric acid excreted and, again, an energy input is involved [41]. Thus, elevated deamination of amino acids coupled with limited ammonia detoxification would result in higher circulating ammonia concentrations, or hyperammonaemia.

**Fig. 2:**
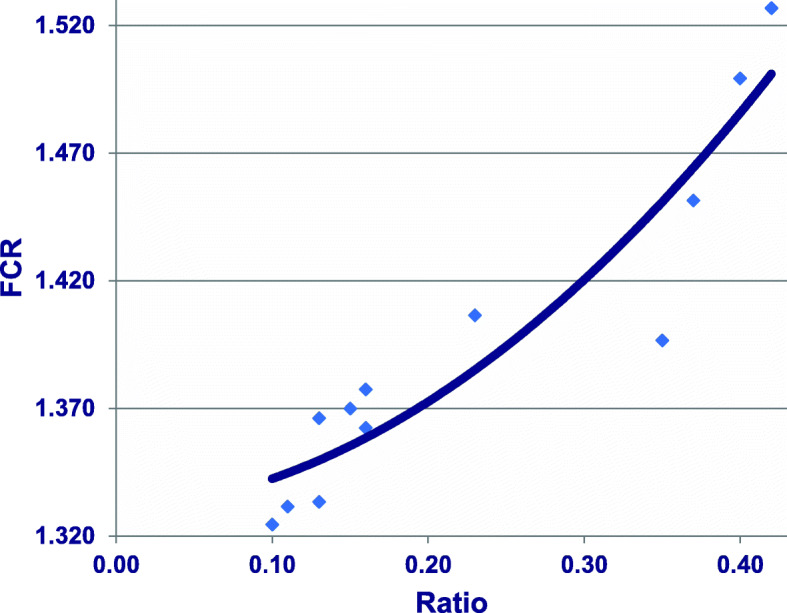
Quadratic relationship (*r* = 0.935; *P* = 0.00009) between ratio of ammonia-N to [ammonia-N plus uric acid-N] in excreta and FCR from 7 to 21 days post-hatch where y = 1.331 + 0.022×ratio + 0.910×ratio^2^. Adapted from Hofmann et al. [42]

Hofmann et al. [42] determined ratios of ammonia-N to [ammonia-N plus uric acid-N] in excreta expressed in mg/day in birds offered diets with three levels of CP and four levels of glycine equivalents from 7 to 21 days post-hatch. It may be calculated from [42] that these ratios were quadratically related to FCR (*r* = 0.935; *P* < 0.0001). As shown in Fig. 2, as ratios of ammonia-N to [ammonia-N plus uric acid-N] expanded there was a deterioration in FCR. This suggests that increasing levels of ammonia-N in excreta, presumably arising from deamination of amino acids, is indicative of poor protein utilisation, which is refected in compromised feed conversion efficiency. Also, it is possible to deduce the proportion of dietary glycine that was involved in uric acid synthesis via the Krebs uric acid cycle on a molar basis from ths study. Across the 12 diets, the mean proportion of dietary glycine utilised in the uric acid cycle was 34.5%; however, individual proportions ranged widely from 14.9% to 66.9%. Clearly, substantial, but highly variable, proportions of dietary glycine are demanded by the Krebs uric acid cycle and, consequently, the most appropriate dietary levels of glycine (or glycine equivalents) will oscillate. Given these outcomes, we are now in the process of determining concentrations of uric acid in excreta retrospectively, as they should prove instructive.

**Fig. 3:**
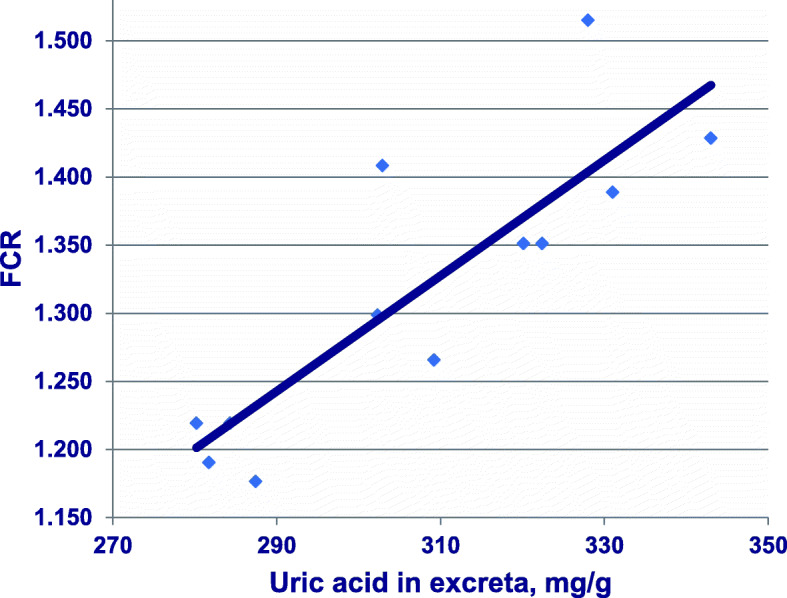
Linear relationship (*r* = 0.850; *P* = 0.0005) between concentrations of uric acid in excreta (mg/g, DM basis) and FCR. Adapted from Donsbough et al. [43], Experiments 2, 3 and 5

Earlier, Donsbough et al. [43] determined uric acid concentrations in excreta of birds offered amino acid-adequate, 212 g/kg CP, maize-soy diets without and with additional methionine and glycine. Instructively, there was a linear relationship (*r* = 0.850; *P* < 0.001) between increasing uric acid concentrations and deteriorating FCR when the outcomes of Experiments 2, 3 and 5 are considered collectively, as shown in Fig. 3. In this study, concentrations of uric acid in excreta appeared to be a more indicative parameter than concentrations of ammonia, uric acid and urea in plasma. Both these outcomes [42, 43] are consistent with the proposal that excess ammonia generated by deamination of surplus amino acids, possibly compounded by inadequate detoxification of ammonia, is negatively impacting broiler growth performance. The more accurate identification of amino acid requirements in broiler chickens offered reduced-CP diets, as opposed to standard diets, should curb amino acid imbalances and, in turn, the costs of deamination.

## Maize versus wheat

Maize was shown to be superior to wheat as the basis of reduced-CP diets in Chrystal et al. (Chrystal PV, Greenhalgh S, McInerney BV, McQuade LR, Selle PH, Liu SY: Maize-based diets are more conducive to crude protein reductions than wheat-based diets for broiler chickens, submitted). Male Ross 308 chicks were offered 222 and 165 g/kg CP diets based on either maize or wheat from 7 to 35 days post-hatch; the diets did not contain any exogenous enzymes. On standard diets (222 g/kg CP), wheat significantly outperformed maize-based diets by 8.54% (2403 versus 2214 g/bird) in weight gain and both feed grains supported an FCR of 1.453. The growth performance of these birds exceeded 2019 Aviagen performance objectives. In contrast, on reduced-CP diets (165 g/kg CP), the maize-based birds outperformed their wheat-based counterparts by 53.0% (2370 versus 1549 g/bird) in weight gain, 22.4% (3481 versus 2843 g/bird) in feed intake and by 19.9% (1.473 versus 1.840) in FCR. In this study (Chrystal PV, Greenhalgh S, McInerney BV, McQuade LR, Selle PH, Liu SY. Maize-based diets are more conducive to crude protein reductions than wheat-based diets for broiler chickens. submitted), 165 g/kg CP wheat-based diets contained more non-bound amino acids (49.4 versus 38.5 g/kg), but less soybean meal (48 versus 113 g/kg) than maize-based diets and both factors may have exacerbated amino acid imbalances. It is tempting to speculate that excessive ammonia levels triggered the inadequate performance of the birds offered wheat-based diets.

In a Hungarian study, Petrilla et al. [44] offered broiler chickens either maize- or wheat-based starter, grower and finisher diets with average CP contents of 207 or 176 g/kg and determined uric acid plasma concentrations at 7, 21 and 42 days post-hatch. Instructively, average uric acid plasma concentrations were 13.6% (334 versus 264 μmol/L) higher in birds offered the high protein wheat-based diets and 26.0% (291 versus 231 μmol/L) higher in birds offered the low protein diets than their maize-based counterparts. This outcome may be indicative of the superiority of maize in this context.

## Starch digestive dynamics

The digestion rates of maize starch and wheat starch may be different in broiler chickens and the inclusion of some slowly digestible starch in broiler diets has been shown to be beneficial [45]. In this study, purified starch from either wheat (rapid) or peas (slow) was evaluated where a 75:25 blend of rapid:slow starch supported the best feed conversion efficiency. It has been suggested that slowly digestible starch may spare amino acids from catabolism in the gut mucosa and enhance their post-enteral availability [46]. The starch digestion rate of wheat (0.035/min) was twice as fast as maize (0.017/min) under in vitro conditions [47]. However, it appears that starch digestion rates in broiler chickens do not differ to the same extent, although starch digestion rates in broiler chickens offered four wheat-based diets were 36.0% (0.117/min versus 0.086/min) numerically faster than birds offered two equivalent maize-based diets (Selle PH, Moss AF, Khoddami A, Chrystal PV, Liu SY: Starch digestion rates in multiple samples of commonly used feed grains in diets for broiler chickens, submitted).

In theory, starch digestive dynamics will dictate the rate at which glucose is absorbed and triggers pancreatic insulin secretions. Slowly digestible starch may promote a more sustained insulin response and more net protein deposition in poultry [48]; however, the physiological roles of insulin in chickens still require elucidation and they are usually considered to be minor [49]. Poultry have high circulating glucose levels and are resistant to insulin and appear to lack the insulin-responsive glucose transporter GLUT-4 [50]. However, GLUT-12, a new glucose transporter in broiler chickens that may be analogous to GLUT-4 in mammals has been identified [51]. Interestingly, Kulcsar et al. [52] reported that plasma insulin concentrations were significantly higher in birds offered maize-based diets than their wheat-based counterparts which is possibly indicative of a sustained insulin response stemming from slower maize starch digestion. Thus, the axis of starch-glucose-insulin in poultry demands clarification as considerable importance is placed on this physiological cascade in pigs [53] and both insulin and amino acids regulate skeletal protein synthesis in young pigs [54].

**Fig. 4:**
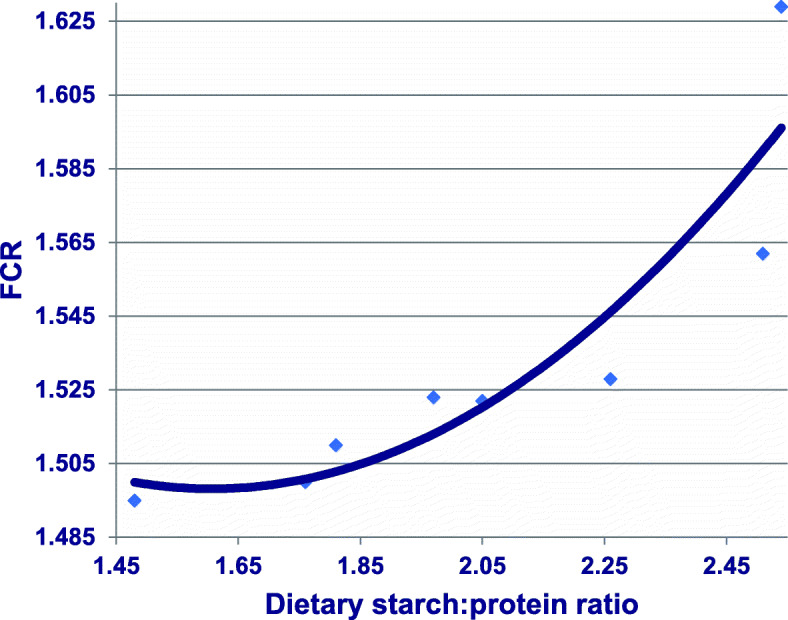
Quadratic relationship (*r* = 0.907; *P* = 0.013) between analysed dietary starch:protein ratios and FCR from 7 to 35 days post-hatch where y = 1.788–0.361×ratio + 0.122×ratio^2^. Adapted from Chrystal et al. [12, 13]

Condensing or ‘capping’ the expansion, of dietary starch:protein ratios in reduced-CP diets may be beneficial. FCR deteriorated in a quadratic manner (*r* = 0.907; *P* = 0.013) in response to expanding dietary starch: protein ratios (analysed) in combined data taken from two studies [14, 15], as shown in Fig. 4. Therefore, the strategy of ‘capping’ dietary starch:protein ratios in wheat-based, reduced-CP diets was evaluated [11] and it did display some promise. Broiler chicks were offered 197.5 g/kg CP diets with dietary starch:protein ratios of either 1.97 or 1.63 from 7 to 35 days post-hatch. Diets with the narrower dietary starch:protein ratio supported better growth performance with significant improvements of 10.4% (2161 versus 1958 g/bird) in weight gain, 3.10% (3492 versus 3387 g/bird) in feed intake and a numerical improvement of 4.04% (1.616 versus 1.684) in FCR. The digestion rate and source of dietary starch has been shown to influence the transition of amino acids across the gut mucosa and entry into the portal circulation in pigs [55, 56], but the importance of this in poultry is not known. Condensing starch:protein ratios in reduced-CP diets is a straightforward concept but its application may be problematical in practice. Partially or entirely substituting soybean meal with feedstuffs containing lesser protein concentrations, including canola meal and other seed meals, may erode the ‘protein quality’ of the diet. However, the substitution of soybean meal (475 g/kg CP) with properly processed full-fat soy (360 g/kg CP) should both maintain dietary protein quality while limiting dietary starch concentrations. Dietary inclusions of field peas (*Pisum sativum*) also merit consideration as their protein contents range from 202 to 267 g/kg and starch from 416 to 475 g/kg [57]. Thus, field peas contain considerably less protein than soybean meal, but their starch content is slowly digestible [58], which may be advantageous. It is our intention to evaluate reduced-CP diets with condensed starch:protein ratios and it is possible that moderate increases in dietary starch may be tolerated by broiler chickens.

## Glycine, serine and glycine equivalents

**Fig. 5:**
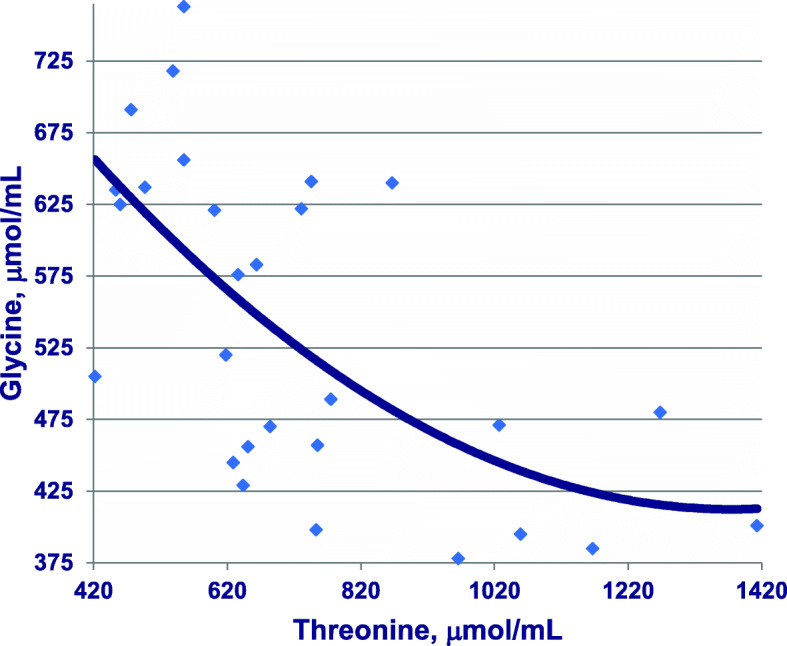
Quadratic relationship (*r* = 0.632; *P* = 0.0017) between free systemic plasma concentrations of threonine and glycine in birds offered maize-based diets with four crude protein concentrations where y = 920.01–0.738×Thr + 0.000268×Thr^2^. Adapted from Chrystal et al. [12]

Glycine supplementation of reduced-CP diets has been shown to support very satisfactory growth performance of broiler chickens by Dean et al. [59]. Dietary glycine plus serine concentrations may be expressed as glycine equivalents [one glycine equivalent (g/kg) equals the sum of glycine plus 0.7143× serine] because there are interconversions between glycine and serine in poultry [60]. In theory, threonine can be metabolised to glycine [61], which is potentially important. However, elevations in threonine concentrations were quadratically related (*r* = 0.632: *P* < 0.001) to declining glycine plasma levels in [13], as illustrated in Fig. 5. This outcome indicates that threonine may not be metabolised to glycine in practice, which agrees with the opinion expressed by D’Mello [62].

A considerable amount of data relating to glycine and serine in the context of reduced-CP broiler diets has been generated by Hohenheim University [42, 63–65] as reflected in the Siegert and Rodehutscord [66] review. These researchers proposed that glycine equivalents are the first-limiting nonessential amino acid in poultry diets and their inclusions will permit more robust reductions in dietary CP. Dietary glycine equivalent inclusions from 11 to 20 g/kg in diets for young broiler chicks were recommended. Justification for this wide range of recommendations was based on variations in dietary concentrations of cysteine and the precursors, threonine and choline. Interestingly, it was suggested that urinary nitrogen excretion may be a major factor in the magnitude of responses to dietary glycine equivalents because of their involvement in uric acid formation [66].

As investigated by Corzo et al. [67], the interactive threonine-glycine-serine axis in reduced-CP diets merits attention. Increased dietary inclusions of threonine (7.15 to 8.25 g/kg) and glycine equivalents (8.37 to 12.70 g/kg), individually and in tandem, were evaluated in a 163 g/kg CP broiler diet by Chrystal et al. [15]. The analysed CP concentrations of the four diets ranged from 158 to 161, 161 and 163 g/kg. The individual additions did not influence growth performance to any extent; however, in tandem these additions increased weight gain by 7.82% (2150 versus 1994 g/bird), improved FCR by 2.69% (1.520 versus 1.562) and reduced relative fat-pad weights by 12.5% (11.65 versus 13.31 g/kg). The responses in weight gain (*P* < 0.025) and relative fat-pad weights (*P* < 0.005) were significant from pair-wise comparisons. It is difficult to account for these arguably synergistic responses as increased threonine could be expected to curb responses to glycine equivalents rather than augment them. However, it is noteworthy that the tandem additions of threonine and glycine equivalents increased mean distal ileal digestibility coefficients of 16 amino acids by 7.99% (0.838 versus 0.776; *P* < 0.01) and distal ileal starch:protein disappearance rate ratios were significantly compressed from 3.23 to 2.64 as a consequence. This outcome may have contributed to the unanticipated responses recorded; however, it does appear that the axis of threonine, glycine and serine in reduced-CP diets merits closer attention.

## Threonine

Threonine is considered the third limiting amino acid in diets for broiler chickens and non-bound threonine became commercially available in the 1980’s. The role of threonine in poultry nutrition has been thoroughly reviewed [68, 69]. In an interesting study [70], rats were offered diets in which amino acids were provided as either protein-bound form as casein or as an equivalent blend of non-bound amino acids. After a 60 min post-prandial period, free threonine plasma concentrations increased by 35.3% (422.0 versus 312.0 μmol/L) in rats offered the casein diet but by 61.1% (767.7 versus 473.7 μmol/L) in diets containing the amino acid blend. This outcome illustrates the inherent differences in non-bound versus protein-bound amino acids as increases in 60 min post-prandial plasma concentrations were observed for the majority of amino acids when offered as a blend. The researchers attributed this outcome to the possibility that casein-bound amino acids supported better whole-body protein homeostasis than non-bound amino acids due to slower intestinal uptake rates.

Intriguingly, pronounced elevations or ‘spikes’ in free threonine plasma concentrations have been observed in broiler chickens pursuant to reductions in dietary CP. The possible underlying mechanisms have been considered as elevated threonine plasma levels may be indicative of poor growth performance of birds offered reduced-CP diets. Fancher and Jensen [71] reported that free threonine plasma concentrations increased by an average of 124% (1959 versus 876 nmol/mL) following the transition from one 183 g/kg CP diet to three 159 g/kg CP diets in female broilers. The three 159 g/kg CP diets contained 1.3 g/kg non-bound threonine and analysed dietary threonine concentrations were 7.2 g/kg across all diets. In contrast, mean plasma concentrations of the balance of 12 amino acids assessed declined by 7.01% (461 versus 428 nmol/mL).

Importantly, similar spikes in systemic plasma threonine concentrations have been consistently observed more recently following reductions in dietary CP levels in maize-based [13–15] (Chrystal PV, Greenhalgh S, McInerney BV, McQuade LR, Selle PH, Liu SY: Maize-based diets are more conducive to crude protein reductions than wheat-based diets for broiler chickens, submitted) and wheat-based diets [10, 11] (Chrystal PV, Greenhalgh S, McInerney BV, McQuade LR, Selle PH, Liu SY: Maize-based diets are more conducive to crude protein reductions than wheat-based diets for broiler chickens, submitted). There was an average reduction in dietary CP of 48.0 g/kg (166.3 versus 213.7 g/kg) across these seven studies when high and low CP dietary levels are compared. These CP reductions generated an average increase of 68.6% (907 versus 538 mmol/L) in free threonine plasma concentrations from 22 observations. Moreover, there is a quadratic relationship (*r* = 0.831; *P* < 0.0001) between threonine plasma concentrations and FCR. The regression equation predicts that once threonine plasma levels exceed 544 mmol/L then FCR begin to deteriorate. This does not necessarily suggest that threonine is compromising FCR; nevertheless, the genesis, purpose and relevance of elevated threonine plasma levels in broiler chickens offered reduced-crude protein diets should be addressed.

Elevated plasma levels cannot result from biosynthesis as threonine is an essential amino acid [72]; therefore, the genesis of elevated threonine levels is likely to be the diminution of threonine catabolism. Theoretically, threonine may be catalysed by three separate pathways. Threonine dehydratase (TH) degrades threonine to α-ketobutyrate; threonine aldolase (TA) to acetaldehyde and glycine and threonine-3-dehydrogeanse (TDH) to acetyl-CoA and glycine [73]. Threonine degradation has changed during the evolution of vertebrates as threonine is mainly degraded by TDH in birds; whereas, in mammals it is degraded by TH [74]. Indeed, hepatic TDH activity (88%) is dominant in avian species (Japanese quail), unlike mammals where TH (93%) is dominant in rats [75].

Several investigations into TDH activity in poultry have been completed [76–82] where the impacts of dietary protein and amino acid concentrations and threonine imbalances have been assessed but the outcomes have been inconclusive. This is reflected in the Davis and Austic [77] suggestion that hepatic TDH activity is influenced by dietary protein levels or other amino acids to a greater extent than by threonine itself. However, Yuan and Austic [79] reported that lowering dietary CP from 320 to 230 g/kg reduced total TDH activity in hepatic mitochondria of chickens by 48.3% (18.3 versus 35.4 units), which could be expected to elevate free threonine plasma concentrations. Thus, the likelihood is that principally hepatic TDH activity is being down-regulated to trigger spikes in free threonine plasma concentrations in broiler chickens offered reduced-CP diets.

The catabolism of ketogenic amino acids, and specifically threonine by TDH, generates acetyl-CoA, which is a central metabolic intermediate capable of influencing the activity of numerous enzymes [83]. Instructively, Guerranti et al. [84] investigated the inhibition of hepatic TDH activity in a study in rats with a focus on fatty acids. These researchers concluded that acetyl-CoA and its derivatives depressed TDH activity by selective feedback inhibition as acetyl-CoA is a major end-product of threonine catabolism. Typically, fat concentrations are decreased in reduced-CP diets; alternatively, starch, and potentially glucose, concentrations and dietary starch:protein ratios are increased. Pivotally, glucose may be metabolised to generate acetyl-CoA [85] and Kaempfer et al. [86] determined the fraction of acetyl-CoA that is derived from glucose in the liver, which are regulated by dietary factors, including carbohydrates. Therefore, elevated acetyl-CoA concentrations derived from relatively high starch/glucose levels in birds offered reduced-CP diets could inhibit or down-regulate TDH activity to generate elevated free threonine plasma concentrations.

**Fig. 6:**
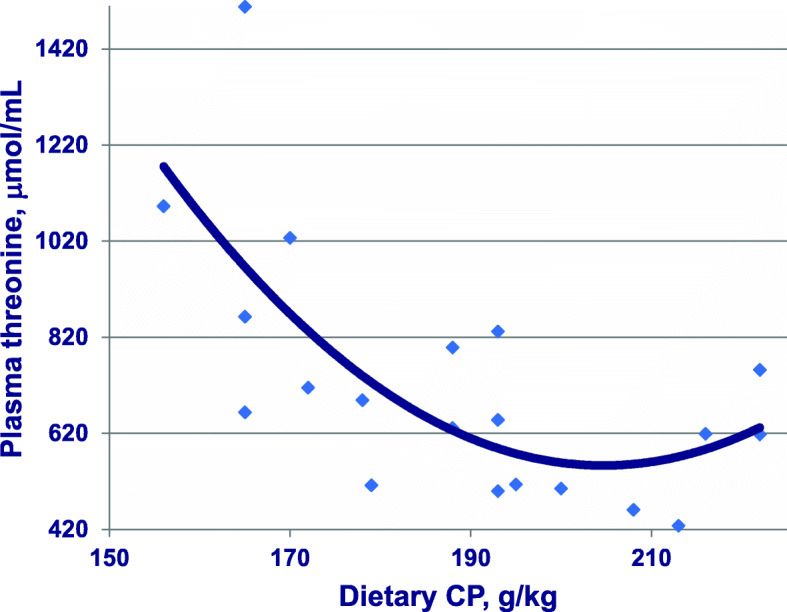
Quadratic relationship (*r* = 0.702; *P* = 0.003) between dietary CP concentrations and free threonine plasma levels across five studies where y = 11,557–107.5×CP + 0.263×CP^2^. Adapted from Moss et al. [8]; Chrystal et al. [11–14]

**Fig. 7:**
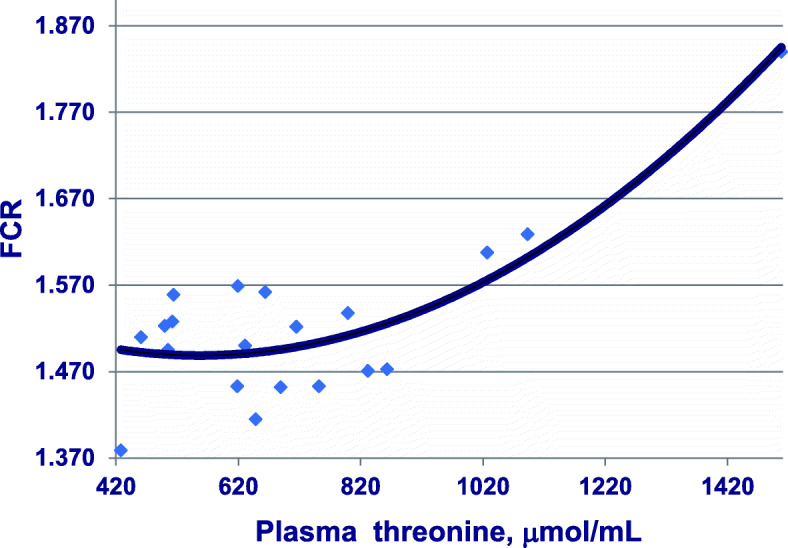
Quadratic relationship (*r* = 0.841; *P* = 0.00003) between free threonine plasma levels and FCR across five studies where y = 1.611–0.004×Thr + 0.0000004×Thr^2^. Adapted from Moss et al. [8]; Chrystal et al. [11–14]

A series of five reduced-CP diet feeding studies have been completed [10–15] (Chrystal PV, Greenhalgh S, McInerney BV, McQuade LR, Selle PH, Liu SY: Maize-based diets are more conducive to crude protein reductions than wheat-based diets for broiler chickens, submitted) in which both dietary starch and protein concentrations were determined and where dietary CP contents were reduced in step-wise gradations from an average of 213.5 to 166.5 CP g/kg. There was a corresponding increase of 34% (417 versus 312 g/kg) in analysed dietary starch concentrations and an expansion from 1.48 to 2.48 in analysed dietary starch:protein ratio across these five studies. A quadratic relationship (*r* = 0.702; *P* < 0.005) from 20 observations between dietary CP and threonine plasma levels was detected in which threonine begins to escalate once CP is reduced below 204.4 g/kg, as shown in Fig. 6. Also, there were significant linear relationships between analysed dietary starch concentrations (*r* = 0.522; *P* < 0.025) and analysed dietary starch:protein ratios (*r* = 0.623; *P* < 0.005) with threonine plasma levels. Finally, there is a quadratic relationship (*r* = 0.841; *P* < 0.0001) between threonine plasma levels and FCR such that high threonine levels are associated with inferior feed conversion efficiency, as shown in Fig. 7. These relationships do not provide validation; however, they are entirely consistent with the principle that acetyl-CoA derived from starch and glucose is down-regulating TDH activity and generating elevated threonine plasma levels in birds offered reduced-CP diets. That both increasing dietary starch and expanding dietary starch:protein ratios are associated with threonine spikes, which accords with the premise. Any differences in starch digestion rates across the various feed grains will influence glucose intestinal uptakes [87], and potentially, the rates at which acetyl-CoA is derived from glucose. This suggests that slowly digestible starch sources may be beneficial from this standpoint. It should prove instructive to determine the effects of dietary CP reductions on threonine plasma levels allied to analysed hepatic concentrations of TDH [88] and acetyl-CoA [89]. Further investigations of this nature are clearly justified and may well accelerate progress towards efficacious reduced-CP diets.

## Summary

In conclusion, every endeavour should be made to harness the potential of reduced-CP diets to facilitate sustainable production of chicken-meat. The more accurate identification of amino acid requirements for broiler chickens offered reduced-CP diets is an obvious objective if amino acid imbalances are to be attenuated. Nevertheless, it is problematic if these requirements can be equally met by non-bound, as opposed to protein-bound, amino acids, which may be further complicated by oligopeptides. The strategy of condensing or capping dietary starch:protein ratios appears promising and merits a closer examination. Maize has been found superior to wheat as the basis of reduced-CP broiler diets, which is an intriguing conundrum. However, a convincing explanation could prove highly instructive as it may involve dietary starch:protein ratios, digestive dynamics of starch and protein and even the starch-glucose-insulin axis. If progress in this and other areas can be realised, then the prospect of reduced-CP diets contributing to sustainable chicken-meat production becomes increasingly real.

## Data Availability

This review is based on published data, which is available.

## Abbreviations

CP: Crude protein
DM: Dry matter
FCR: Feed conversion ratio
N: Nitrogen
Na: Sodium
TA: Threonine aldolase
TDH: Threonine dehydrogenase
TH: Threonine dehydratase
